# Diverse *Mycena* Fungi and Their Potential for *Gastrodia elata* Germination

**DOI:** 10.4014/jmb.2401.01009

**Published:** 2024-04-29

**Authors:** Xiao-Han Jin, Yu-Chuan Wang, Dong Li, Yu Li, Hai-Yan He, Han-Bo Zhang

**Affiliations:** 1 State Key Laboratory Conservation and Utilization of Bio-Resources in Yunnan, Kunming, P.R. China; 2 School of Ecology and Environmental Science, Yunnan University, Kunming, P.R. China; 3 Gastrodia Tuber Research Institute of Zhaotong, P.R. China; 4 The Agriculture and Life Sciences College, Zhaotong University, Zhaotong, P.R. China; 5 Yunnan Key Laboratory of *Gastrodia elata* and Fungus Symbiotic Biology, Zhaotong, P.R. China

**Keywords:** *Gastrodia elata* variety, *Mycena*, phylogenetic signal, geographical distribution, germination

## Abstract

It remains to be determined whether there is a geographical distribution pattern and phylogenetic signals for the *Mycena* strains with seed germination of the orchid plant *Gastrodia elata*. This study analyzed the community composition and phylogenetics of 72 *Mycena* strains associated with *G. elata* varieties (*G. elata*. f. *glauca* and *G. elata*. f. *viridis*) using multiple gene fragments (ITS+nLSU+SSU). We found that (1) these diverse *Mycena* phylogenetically belong to the Basidiospore amyloid group. (2) There is a phylogenetic signal of *Mycena* for germination of *G. elata*. Those strains phylogenetically close to *M. abramsii*, *M. polygramma*, and an unclassified *Mycena* had significantly higher germination rates than those to *M. citrinomarginata*. (3) The *Mycena* distribution depends on geographic site and *G. elata* variety. Both unclassified *Mycena* group 1 and the *M. abramsii* group were dominant for the two varieties of *G. elata*; in contrast, the *M. citrinomarginata* group was dominant in *G. elata* f. *glauca* but absent in *G. elata* f. *viridis*. Our results indicate that the community composition of numerous *Mycena* resources in the Zhaotong area varies by geographical location and *G. elata* variety. Importantly, our results also indicate that *Mycena*’s phylogenetic status is correlated with its germination rate.

## Introduction

The fungi of the genus *Mycena* (Pers.) Roussel belong to the phylum Basidiomycota, class Agaricomycetes, order Agaricales, and family Mycenaceae. The genus is species-rich and cosmopolitan. Several monographs of *Mycena* from different continents have been published over the years [1-5]. According to the tenth edition of Ainsworth & Bisby’s Dictionary of the Fungi, *Mycena* is one of the most diverse genera in Agaricales, comprising more than 500 described species worldwide [5]. As of 2023, Index Fungorum lists 2412 records for this genus. Most species of *Mycena* are saprotrophic fungi that decompose litter and contribute to the forest ecosystem [6, 7]. However, some species have symbiotic or parasitic interactions with plants. For example, *Mycena galopus* forms beneficial associations with *Vaccinium corymbosum* [8]; *M. citrinomarginata* colonizes the roots of Festuca roemeri [9]; and *Mycena citricolor* infects coffee trees and causes American leaf spot disease [10].

The Orchidaceae family is exceptionally diverse and widespread [11], comprising over 750 genera and approximately 27000 species, and is the most species-rich, accounting for approximately 10% of the world’s flowering plants [12, 13]. Orchid seeds are very small (dust-like), lack endosperm and have limited nutrition for germination, so they depend on certain fungi to supply them with nutrients. These plants are known as “initially mycoheterotrophic” plants [14-17]. All orchids are at least initially mycoheterotrophic [18, 19] and depend entirely on orchid mycorrhizal fungi (OMFs) for seed germination and protocorm formation [20, 21]. Subsequently, most orchids develop green leaves and perform photosynthesis (partially mycoheterotrophic type), whereas more than 200 orchid species never photosynthesize and rely entirely on fungal nutrition for growth (fully mycoheterotrophic type) [11, 22].

*Gastrodia elata* Bl. belongs to the fully mycoheterotrophic type and has important medicinal and edible value. It is widely used in China, Korea and Japan [23]. Each of its capsules produces tens of thousands of seeds [24], but they lack nutrition and require symbiosis with *Mycena* fungi to germinate and form protocorms. Subsequent nutritional growth depends on symbiosis with another fungus, *Armillaria mellea*, to complete the whole life cycle [25]. Although related cultivation technology and practices have achieved success, natural *G. elata* populations are still facing threats and are rated vulnerable on the IUCN Red List (IUCN 2022).

Currently, it is generally believed that *G. elata* seeds can only germinate in symbiosis with *Mycena* [26]. In the 1980s, Xu and Guo [27] were the first to successfully induce the fruiting body of a fungal strain for *G. elata* seed germination, GSF-8104, which was identified as *Mycena osmundicola*. In the 1990s, Guo and Fan *et al*. isolated three strains from the roots of 45 orchid species that could symbiotically enhance the germination of *G. elata* seeds. These strains were subsequently identified and named *Mycena orchidicola* [28], *Mycena anoectochila* [29] and *Mycena dendrobii* [30]. Since then, it has been rare to report new *Mycena* strains with the capacity for *G. elata* germination because *Mycena* are weakly saprophytic and have slow growth and low culturability. Until 2020, there were new reports on *G. elata* seed germination fungi, where two strains belonging to *Mycena citrinomarginata* and *Mycena purpureofusa* could germinate *G. elata* seeds [31]. In 2022, it was demonstrated that a new *Mycena* species, *Mycena subpiligera*, discovered in subtropical regions of China, could promote *G. elata* seed germination [32]. These studies indicate that research on the germinating fungi of *G. elata* seeds has not been interrupted, but few strains have been obtained, especially in Zhaotong, China, the main production area of *G. elata*, with scarce germinating fungi resources.

**Fig. 1:**
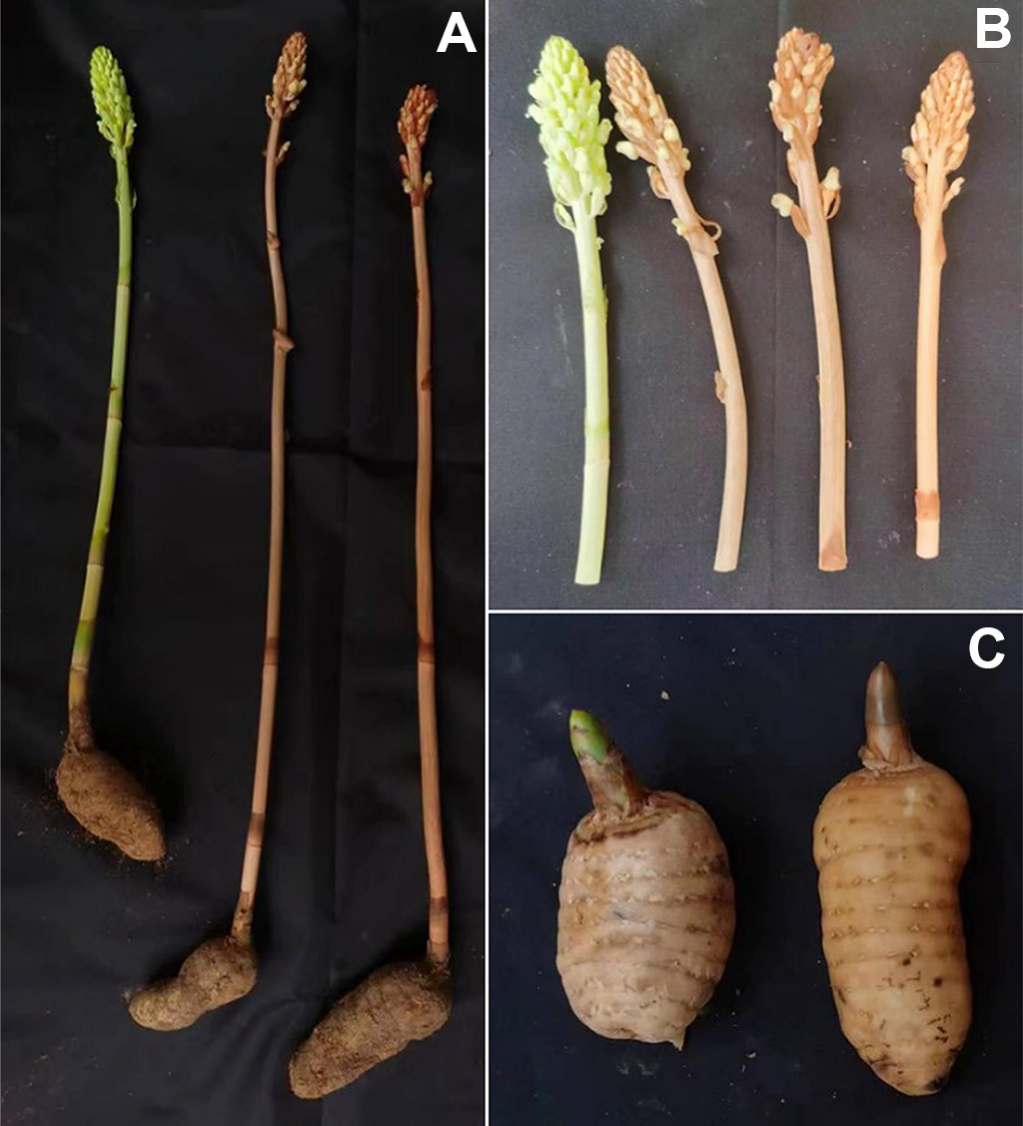
Morphology of *G. elata*. f. *viridis* and *G. elata*. f. *glauca*. *G. elata*. f. *viridis* is shown on the far left, and *G. elata*. f. *glauca* is shown on the right. Fig. a depicts the morphological characteristics of the upright stem of *G. elata* after bolting; Fig. b illustrates the morphological characteristics of the tubers of *G. elata*; Fig. c demonstrates the morphological characteristics of the flowers of *G. elata*. *G. elata*. f. *glauca* has a plant height of 1.5-2 meters or more; a gray‒brown stem with white longitudinal stripes (**A**) blue‒green flowers (**B**) an elliptical or ovoid tuber, up to 15 centimeters or longer, with a maximum weight of 0.8 kilograms and a water content of 60-70% (**C**). *G. elata*. f. *viridis* has a plant height of 1-1.5 meters; a pale blue‒green stem (**A**) pale blue‒green or white flowers, which are rare (**B**) and an elliptical or inverted cone-shaped tuber, with a maximum weight of 0.6 kilograms and a water content of approximately 70% (**C**) [73].

Nonetheless, *Mycena* is a diverse genus of fungi in China, and 76 names of *Mycena* have been recorded, including 6 new species and 16 new records from China [33, 34]. Recently, Na [35] used the ITS+nLSU+SSU multigene sequencing method to reconstruct the phylogenetic status of *Mycena* on a large scale. The two main branches of the genus are characterized by starch-like and nonstarch-like spores, divided into 7 clades, involving 56 taxonomic units of 23 groups of Mycena. Currently, it is unclear whether there is a phylogenetic relationship between *Mycena* species and the seed germination of *G. elata*. Moreover, it also remains to be determined whether different strains within the same species vary in their germination efficiency for different *G. elata* varieties. There are two varieties of *G. elata* with different geographical distributions in Zhaotong city, the most important *G. elata* production area in China, namely, *G. elata* Bl. f. *glauca* and *G. elata* Bl. f. *viridis* (Fig. 1). *G. elata* f. *glauca* is mainly found in Zhaoyang District, Zhenxiong County, Yiliang County and Weixin County, among which the Xiaocaoba *G. elata* in Yiliang County is the most famous, known as the “cloud *G. elata*”. *G. elata* f. *viridis* is mainly found in Qiaojia County, Yanjin County, Daguan County and Yongshan County, among which the Shibanba *G. elata* in Qiaojia County is the most famous, known as the “green gold”. If only some species can induce the germination of a given *G. elata* variety, it is thus expected that the geographic distribution of *Mycena* species with germination potential can determine the geographic distribution of *G. elata* variety and conservation.

In this study, we obtained numerically diverse *Mycena* by using an *in situ* fungal baiting method with different varieties of *G. elata* in natural habitats in Zhaotong, China, and verified the germination rate of these strains on *G. elata* varieties. We also used multigene methods to examine the correlation between the phylogenetic status of *Mycena* and the germination rate of *G. elata*. We aimed to answer the following questions: (1) What is the diversity of *Mycena* species in natural habitats in Zhaotong?(2) Is there a phylogenetic signal of *Mycena* for the germination of *G. elata*? (3) Is there a geographic distribution of *Mycena* and host preference of *Mycena* for the *G. elata* variety?Answering these scientific questions has important theoretical implications for understanding the evolution of *Mycena*-*G. elata* symbiosis, as well as practical guidance for isolating and obtaining novel germination-promoting *Mycena* resources.

## Materials and Methods

### *In situ* Fungal Baiting

In July 2021, we performed an experiment for *in situ* fungal baiting in three main production areas of *G. elata* in Zhaotong, China, including Yiliang (YL), Lianfeng (LF), and Panhe (PH). The baiting site was selected in the natural habitat with the distribution of wild *G. elata* populations. To attract suitable fungi for germination, we mixed local dead Fagaceae leaves with seeds of two varieties of *G. elata*, *G. elata* f. *glauca* and *G. elata* f. *viridis*, and then loaded them into a nylon mesh bag with dimensions of 45 cm (length) × 30 cm (width) and a grid of 1 mm. Nylon bags were buried 3 cm below the ground surface, with a minimum spacing of 1 m between each bag. At least three biological replicates for each variety of seeds were buried in each sample plot. In October 2021, we retrieved the bags buried in YL, and in December 2021, we retrieved the bags buried in LF and PH. These samples were put in a foam box and brought back to the laboratory with an ice pack to maintain a low temperature. The seeds of *G. elata* f. *glauca* and *G. elata* f. *viridis* were purchased from a *G. elata* farmer in Zhaotong, China. *G. elata* tissue samples were harvested from the nylon bags in the laboratory and stored at 4°C until utilization.

### Isolation, DNA Extraction, and PCR Amplification of Endophytic Fungi

Endophytic fungi were isolated from the *G. elata* protocorms collected from the seed baiting bags. The protocorms were extracted and surface-sterilized by soaking in a 0.5% sodium hypochlorite solution for 30 s and then soaking in a 75% ethanol solution for 30 s, rinsed three times with sterile water, and then dried with sterile filter paper. The protocorms were cut into small pieces of approximately 2 mm using a sterile dissecting knife. We placed small pieces of protocorms on different Petri dishes: potato dextrose agar (PDA, potato 200 g/L, dextrose 20 g/L, agar 16 g/L), 1/5 nutrient potato dextrose agar (1/5 PDA, potato 40 g/L, dextrose 4 g/L, agar 16 g/L), wheat bran potato dextrose agar (wheat bran 150 g/L, potato 100 g/L, dextrose 20 g/L, agar 10 g/L), fungal isolation medium (FIM, (Ca(NO_3_)_2_·4H_2_O 0.5 g/L, KH_2_PO_4_ 0.2 g/L, KCl 0.1 g/L, MgSO_4_·7H_2_O 0.1 g/L, yeast extract 0.1 g/L, sucrose 5 g/L, agar 10 g/L) and potato sucrose malt agar (potato 200 g/L, sucrose 20 g/L, malt 10 g/L, agar 16 g/L). We incubated the plates in the dark at 25°C for 3-5 days. Then, we transferred the fungi that grew out from the protocorm pieces to fresh PDA plates until we obtained pure strains.

We extracted genomic DNA from fungal strains using the cetyltrimethylammonium bromide (CTAB) method [36]. The sample was treated with a CTAB buffer solution containing salts and other reagents. This solution breaks down cell membranes, releasing DNA. After heating to precipitate proteins, DNA is extracted by adding alcohol, which causes it to precipitate out of the solution. The precipitated DNA is then purified to remove impurities, resulting in a relatively pure DNA solution suitable for PCR and sequencing. We first sequenced the ITS genes of all isolated strains using primers ITS4 and ITS5 and following the PCR cycling conditions described by White *et al*.[37]. We then amplified the nLSU and SSU regions of the *Mycena* DNA using primers LR0R and LR7 and primers MS1 and MS2, following the PCR cycling conditions described by Hopple and Vilgalys [38] and Ward and Gray [39], respectively. We used a 50 μl system consisting of 25 μl of 2 × PCR Master Mix (Sangon, Shanghai, China), 1 μl of 10 μl of forward primer and reverse primer each, 1 μl of template DNA and 22 μl of sterile ddH_2_O. The PCR products were sequenced by Sanger sequencing at Shanghai Sangon Biotech Co., Ltd. (China).

### Phylogenetic Analysis

We performed OTU division and species identification for all isolated strains using ITS gene sequencing results and then used the combination of ITS, nLSU, and SSU gene fragments to perform OTU division for Mycena. We summarized and checked the orientation of the sequences with SeqMan 7.0.0 (DNAstar 5.0) and aligned, trimmed and concatenated each gene sequence with BioEdit 7.0.4.1 [40]. We assigned the trimmed sequences to OTUs at the unique level and 99% sequence similarity level using Muthur 1.35.1 software [41] and performed taxonomic classification based on the UNITE database [41]. We selected five representative strains of *Mycena* at the 99% similarity level in the combination of ITS, nLSU, and SSU gene fragments to construct the multigene phylogenetic tree.

All gene nucleotide sequences for these five representative strains reported in this study were deposited at GenBank under the accession numbers OR759509–OR759513 for ITS, OR754290–OR754294 for nLSU and OR763347–OR763351 for SSU (Table S1).

In this study, we obtained a total of 72 strains and one commercial *Mycena* J3) × 3 genes = 219 sequences by sequencing three genes (ITS, nLSU, and SSU) for 72 strains and one commercial *Mycena* J3. We also downloaded 70 sequences from GenBank, comprising 41 ITS, 17 nLSU, and 12 SSU sequences. The reference sequence information is presented in Table S2. We constructed a phylogenetic tree for the abovementioned 289 *Mycena* sequences based on the combination of ITS, nLSU, and SSU gene fragments. We also constructed a phylogenetic tree for the 17 *Mycena* sequences from this study based on the same combination of gene fragments. We determined the best nucleotide substitution model for each gene sequence using ModelFinder in PhyloSuite v1.2.2 [42]. We inferred the phylogeny of *Mycena* using Bayesian inference (BI) and maximum likelihood (ML) methods with *Xeromphalina campanella* (Batsch) Kühner & Maire as the outgroup. The BI analysis was performed with MrBayes version 3.2.7 (Sweden) [43]. Markov chain Monte Carlo (MCMC) chains were run for one million generations, sampling every 100th generation until the topological convergence diagnostic was less than 0.01 [44]. ML analysis was performed on MEGA 11 [45] using the GTR+G model. The bootstrap value was set to 1000 in the ML analysis, and the bootstrap values on the nodes were used to assess the reliability of the phylogenetic tree. Topology support values greater than 80% bootstrap support (ML) and 0.9 Bayesian posterior probabilities (BPP) are shown at each branch node. The resulting tree was visualized in Figtree version 1.4.4 (UK).

### Germination Experiment of *Mycena* for *G. elata* Seeds

**Germination test in the lab.** The germination experiment was set up as shown in Fig. S1. Preparation of fungus-treated leaves: Leaves of the Fagaceae family were collected in the wild (Xishan Forest Park, Kunming, China, March, 2022), dried and cut into 2 cm × 2 cm pieces. The leaf pieces were sterilized according to soil sterilization standards (sterilized 3 times at 121°C, each time for 120 min, with a 24-h interval between each sterilization), dried and placed flat on PDA. Fresh *Mycena* hyphae were inoculated around the Fagaceae leaf pieces and incubated until the leaves were fully colonized by the hyphae (15 days). The leaf pieces colonized by the hyphae were placed flat on water agar medium. Then, *G. elata* seeds were evenly distributed on the fungus-treated leaf pieces. We used the commercially utilized strain *Mycena* J3 as a positive control and sprinkled the seeds directly on water agar plates (Fig. S2A), PDA plates (Fig. S2B), and sterile Fagaceae leaves and placed them on water agar plates (Fig. S2C) as a negative control. The plates were incubated at 25°C in the dark. We monitored seed germination under a dissecting microscope using the rupture of the seed coat as the germination criterion (Fig. S2D and S2E). We measured the germination rate in the fourth week after sowing, randomly selected 5 fields (Fig. S2F and S2G) under a dissecting microscope to record the number of germinated seeds and the total number of seeds, and calculated the germination rate. Germination rate (%) = (number of germinated seeds/total number of seeds) × 100%.

**Germination test in the wild.** We chose two *Mycena* strains (YL10 and YL16) with high germination rates to perform a field experiment. The site with sandy loam soils was located in YL, the main production area of *G. elata* in Zhaotong. The fungal bag was prepared according to the production formula of a *Mycena* fungus bag (57%broad-leaved tree leaves, 20% sawdust, 20.45% bran, 1% sucrose, 0.3% potassium dihydrogen phosphate, 0.25%magnesium sulfate, 1% gypsum powder, 65% water content, natural pH) and inoculated after sterilization according to the soil sterilization standard. After one and a half months, the bag was fully colonized by *Mycena* mycelium. The fungal materials were mixed with *G. elata* seeds and loaded into mesh bags with dimensions of 45 cm (length) × 30 cm (width) and a grid of 1 mm. The mesh seed bags were buried 5 cm below the soil surface, with a minimum distance of 1 m between each bag. After five months, they were retrieved, and *G. elata* tissues were sorted out in the laboratory. We counted the number of *G. elata* tissues and calculated the dry weight of each *G. elata* tissue in each bag. We also used commercial *Mycena* J3 as a positive control to verify the germination effect in the wild.

### Data Analysis

We used Shapiro‒Wilk and Kolmogorov‒Smirnov tests to assess the normality of the data and performed independent sample T tests, analysis of variance (ANOVA) and post hoc comparisons if the data were normally distributed or nonparametric analysis if not. In this experiment, we used an independent sample T test to compare the germination rate between *G. elata* f. *glauca* and *G. elata* f. *viridis* for each strain, as well as the germination rate between each tested strain and the control commercial *Mycena* J3. We used one-way analysis of variance and post hoc multiple comparison to analyze the number of *G. elata* tissues and dry weight of single *G. elata* tissue. Among them, based on the homogeneity of data variance, Duncan’s test (homogeneous variance) and Dunnett’s T3 test (heterogeneous variance) were selected in post hoc multiple comparisons. We used nonparametric analysis to compare the germination rate by location, *G. elata* varieties and OTU division. SPSS 22.0 software (SPSS Inc., USA) was used for the normality test, one-way analysis of variance, post hoc multiple comparison and nonparametric Mann‒Whitney U tests. GraphPad Prism 7 software (GraphPad-software Inc., USA) was used to draw charts of fungal composition, germination rate of each strain, germination rate by location, germination rate by *G. elata* varieties, germination rate by OTU division, number of *G. elata* tissues and dry weight of single *G. elata* tissue.

## Results

### Geographic and Host Patterns of the Endophytic Fungal Community

In total, we obtained 280 strains from *G. elata* tissues (Table 1). Based on their ITS sequences, these strains phylogenetically belong to 208 strains of Ascomycetes and 72 strains of Basidiomycetes. The most abundant fungi at the genus level were *Ilyonectria*, *Cadophora*, and *Mycena*, accounting for 41.43%, 28.21%, and 24.29% of the total strains, respectively. Geographically, the fungal community is distinctively distributed (Table 1). The most abundant genus at the YL site was *Mycena*, accounting for 90% of the total isolates. In contrast, the most abundant genus at the LF site was *Ilyonectria*, with 51.49% of the total strains, followed by *Cadophora*, with 35.15% occurrence; similarly, the dominant genera at the PH site were also *Ilyonectria* and *Cadophora*, with 41.38% and 27.59%occurrence, respectively. Meanwhile, these two sites also harbored 11.76% and 10.71% of *Mycena*, respectively. Two varieties, *G. elata* f. *glauca* and *G. elata* f. *viridis*, harbored similar fungal communities, with the most prevalent being *Ilyonectria*, accounting for 43.11% and 34.55% of the total sequence, respectively. The second and third dominant genera were *Mycena* and *Cadophora*, which accounted for 27.11% and 27.11% of the total sequence of *G. elata* f. *glauca*, respectively, and 32.73% and 12.73% of the total sequence of *G. elata* f. *viridis*, respectively (Table 1).

### Phylogenetic Analysis of Multiple Genes in Mycena

**Fig. 2:**
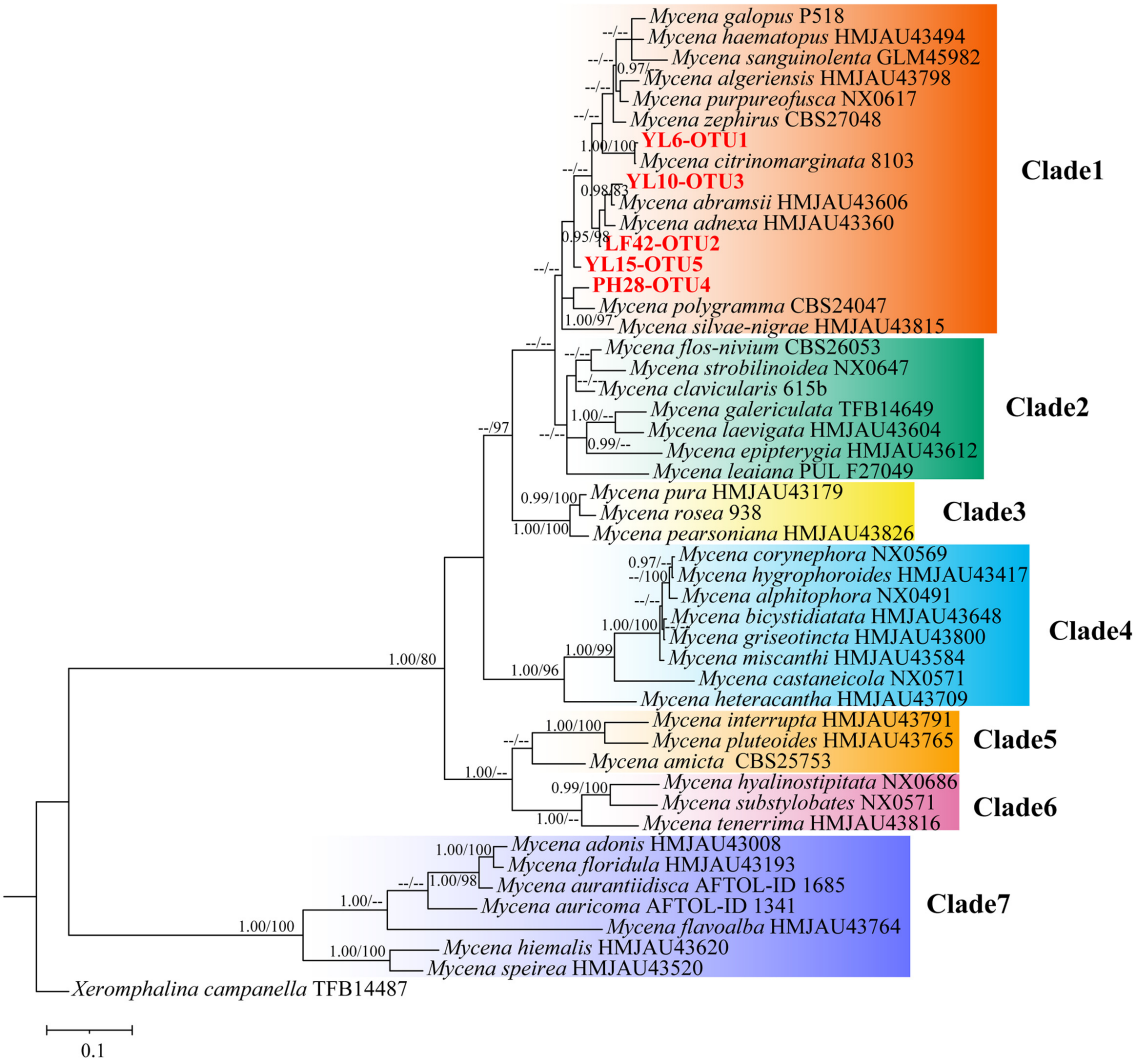
Mycena phylogeny from ITS+nLSU+SSU multi-gene sequences analysis (BPP ≥ 0.95, Bootstrap≥75% on nodes, – otherwise). The sequences of this study are displayed in bold red font, followed by the symbol “-” and the OTU at the 99% similarity level in ITS+nLSU+SSU genes indicating the OTU represented by this sequence. The tree is rooted with *Xerphalina campanella*.

Based on the ITS gene sequences, we identified 72 *Mycena* that were classified into 5 OTUs at the 99% similarity level. Phylogenetic analysis showed that OTU1 and OTU4 were closely related to *M. citrinomarginata* and *M. polygramma*, respectively. OTU2 and OTU3 were sister taxa to *M. adnexa* and *M. abramsii*. OTU5 formed a separate branch (Fig. S3). Therefore, we further constructed a phylogenetic tree of the genus *Mycena* based on the combination of ITS+nLSU+SSU gene fragments (Fig. 2). According to the BI method and ML method, these strains were divided into 7 clades to form two large groups, with stable support rates for each clade, namely, the Basidiospore amyloid group (Clade 1-Clade 6) and Basidiospore nonamyloid group (Clade 7). All of our 72 *Mycena* strains are clustered in Clade 1. Consistent with the phylogenetic tree of ITS genes, OTU1 and OTU4 were closely related to *M. citrinomarginata* and *M. polygramma*, respectively. OTU5 formed a separate branch. However, OTU3 was closely related to *M. abramsii*, while OTU2 was sister to *M. adnexa*, *M. abramsii* and OTU3.

**Fig. 3:**
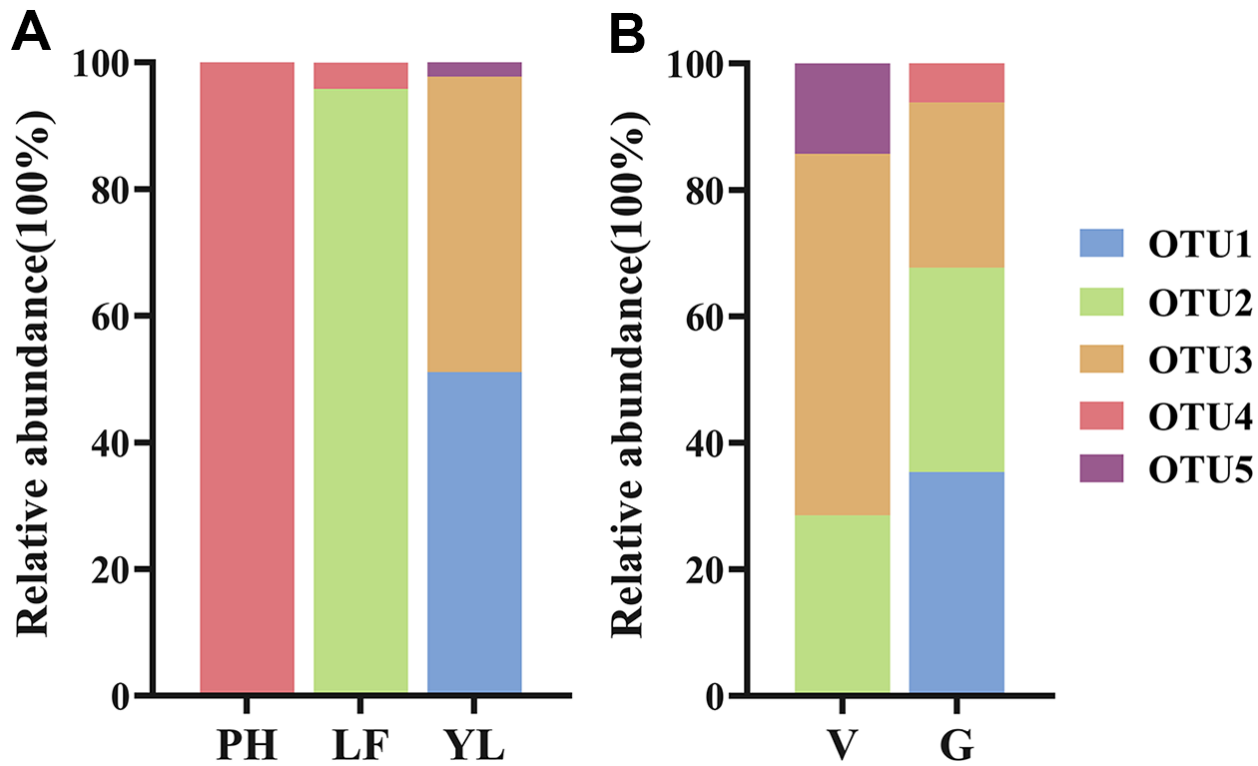
The OTU relative abundance of *Mycena* strains occurring in geographic site (**A**) and *G. elata* variety (**B**). YL, Yiliang; LF, Lianfeng; PH, Panhe. G, *G. elata* f. *glauca*; V, *G. elata* f. *viridis*.

The most abundant species were OTU1 (*M. citrinomarginata* group) and OTU2 (unclassified *Mycena* group 1), each accounting for 31.94% of the total *Mycena* strains, followed by OTU3 (*M. abramsii* group), with an abundance of 29.17% (Table S3). We obtained 45 *Mycena* from the YL site, of which OTU1 (*M. citrinomarginata* group) and OTU3 (*M. abramsii* group) had abundances of 51.11% and 46.67%, respectively; 24 *Mycena* from the LF site, of which OTU2 (unclassified *Mycena* group1) was the dominant species, with an abundance of 95.83%; and only 3 *Mycena* from the PH site, all of which were OTU4 (*M. polygramma* group) (Fig. 3A, Table S3). Regarding the two varieties of *G. elata*, both OTU2 (unclassified *Mycena* group 1) and OTU3 (*M. abramsii* group) were dominant, with abundances of 32.31% and 26.15% in *G. elata* f. *glauca* and 57.14% and 28.57% in *G. elata* f. *viridis*, respectively. In contrast, OTU1 (*M. citrinomarginata* group) occurred at 35.38% in *G. elata* f. *glauca* but at zero in *G. elata* f. *viridis* (Fig. 3B, Table S3). Therefore, the *Mycena* distribution depends on the geographic site and *G. elata* variety.

### Germination Promotion for *G. elata* Seeds

**Fig. 4:**
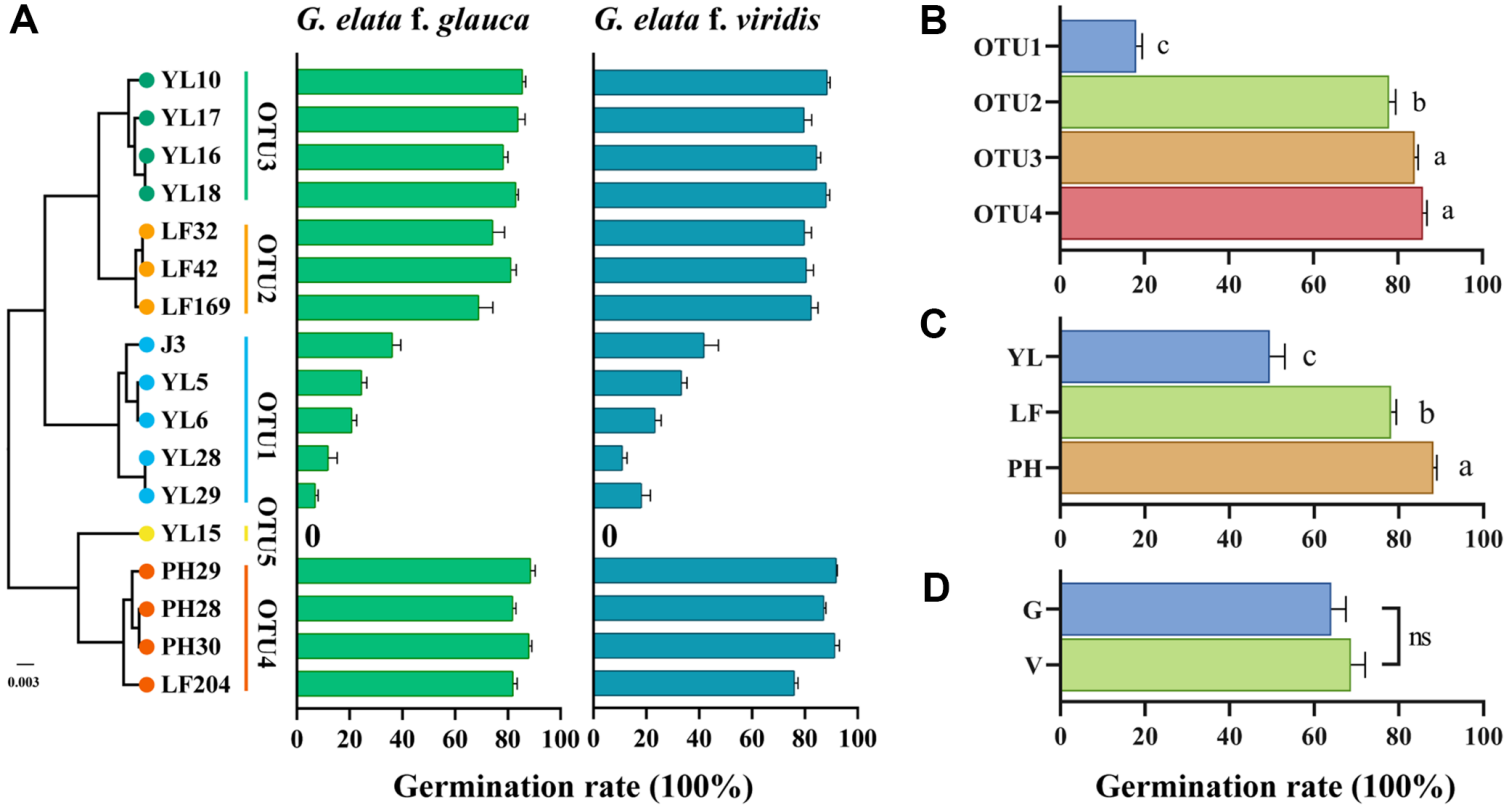
The effect of different *Mycena* on the germination rate of *G. elata* seeds. (**A**) Phylogenetic tree of the ITS+nLSU+SSU genes in this study’s sequences and their correlation with the germination rates of *G. elata* f. *glauca* and *G. elata* f. *viridis*. J3 is the control commercial Mycena. The uppercase letters and numbers in the column where J3 is located represent the *Mycena* strain number. (**B**) Statistical comparison of germination rate by OTU. (**C**) Statistical comparison of germination rate by sample plot (YL, Yiliang; LF, Lianfeng; PH, Panhe). (**D**) Statistical comparison of germination rate by *G. elata* variety (G, *G. elata* f. *glauca*; V, *G. elata* f. *viridis*). Different lowercase letters indicate significant differences (*P*<0.05), and identical lowercase or ns indicate nonsignificant differences (*P* > 0.05). Data are the mean ± SEM, n ≥ 3.

We first tested the germination rate for *G. elata* of 17 *Mycena* strains belonging to five OTUs in the laboratory (Fig. S4). With the exception of YL15 (OTU5), all strains were able to promote *G. elata* germination. Ten strains had significantly higher germination rates for *G. elata* f. *glauca* and eight for *G. elata* f. *viridis* than the positive control (a commercial *Mycena* J3). There was a certain correlation between the phylogenetic status of *Mycena* and the germination rate (Fig. 4A). The germination rates of OTU2, OTU3, and OTU4 were significantly higher than that of OTU1. The germination rates of OTU3 and OTU4 were not significantly different, but both were significantly higher than that of OTU2 (Fig. 4B). The germination rates of the strains were significantly different among the three geographic locations (Fig. 4C). Most strains showed higher germination for *G. elata* f. *viridis* than for *G. elata* f. *glauca* (Fig. S4); however, the average germination rate was not significantly different between *G. elata* f. *viridis* and *G. elata* f. *glauca* (Fig. 4D).

**Fig. 5:**
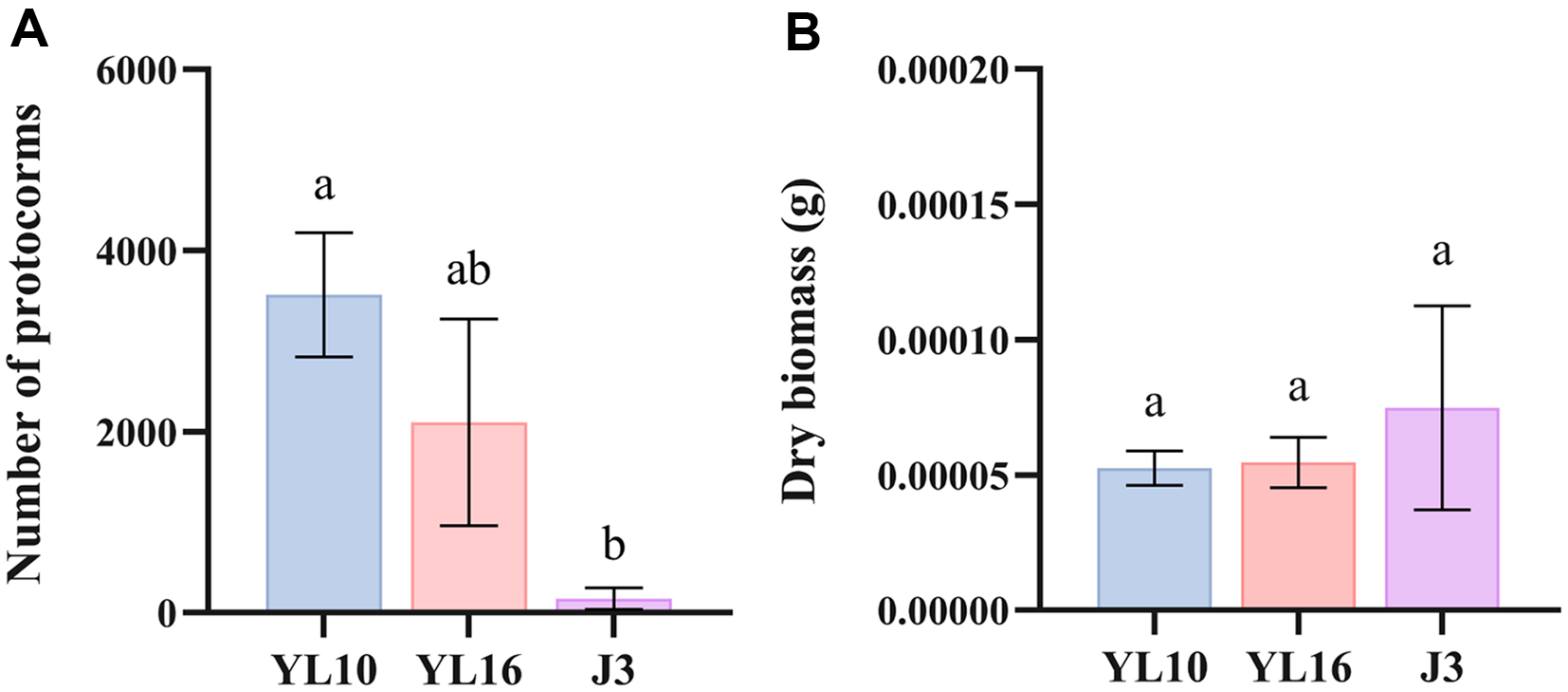
The number of germinated protocorms (**A**) and the dry weight of a single *G. elata* protocorm (**B**) of *Mycena* strains YL10, YL16 and commercial *Mycena* J3 in field experiments. Different letters indicate significant differences (*P* < 0.05, Fig. a for Duncan’s test and Fig. b for Dunnett’s T3 test). Data are the mean ± SEM, n ≥ 3.

In the field germination experiment (Fig. S5), an average of 3511 and 2103 protocorms were harvested for the YL10 and YL16 strains, respectively, whereas only 157 protocorms were harvested for the commercial *Mycena* J3. Although the dry biomass of a single protocorm was not different among the three tested strains, the number of harvested protocorms of YL10 was significantly higher than that of J3 (Fig. 5).

## Discussion

We obtained numerically diverse *Mycena* in natural habitats in Zhaotong, China. Moreover, most *Mycena* strains showed a high capacity for *G. elata* germination, which is why Zhaotong is the main production area of *G. elata* in China. The genus *Mycena* comprises many species that are small and similar in appearance, posing challenges for identification and classification. Microscopic features of their basidiocarps, such as spores, lamellar cystesia, pileipellis and stipitipellis, are usually used for taxonomic purposes [5]. Because it failed to induce basidiocarps for these *Mycena* strains in this study, we had to deduce their phylogenetic position using molecular sequencing data and related literature, and the species were placed in the nearest known taxa. The phylogenetic tree constructed based on ITS+nLUS+SSU multigene analysis is more complete than that constructed based on single-gene analysis. The single-gene trees support the topology of the multigene tree and distinguish well between the two main clades of *Mycena*, namely, the Basidiospore amyloid clade and the Basidiospore nonamyloid clade, which are further divided into 7 clades (Fig. S3). However, in the ITS tree, the group of YL10 in the *Fragilipedes* section is not clear. In the multigene tree, Clade 1, Clade 2, and Clade 3 are sisters to Clade 4, while in the ITS tree, Clade 1, Clade 2, and Clade 3 are sisters to Clade 5 and Clade 6. The support value of Clade 1 in the ITS tree was higher than that in the multigene tree. Multigene analysis has also helped to resolve the phylogenetic relationships of some complex species within *Mycena*, and selecting appropriate gene fragments can better elucidate the divergence of the phylogenetic framework within Mycena. When screening suitable fragments, some species showed severe peak overlap and heterozygosity in ITS sequencing, while nLSU and SSU showed high conservation and good results. The possible new species are strains LF42 and YL15, which form a single branch in both the multigene tree and the ITS tree. Our data suggested that this area is valuable for exploring novel *Mycena* that can induce the germination of *G. elata* seeds.

From a geographical perspective, the compositions of both the total fungal communities and the *Mycena* community were different among the three plots (YL, LF, and PH) (Fig. 2A and 3A). Similarly, some studies have shown that orchids have different mycorrhizal fungal communities in different habitats, and environmental conditions strongly affect their mycorrhizal fungal communities [46, 47]. For example, *Neottia ovata* has different mycorrhizal fungal compositions in grasslands and forests [48]. The same orchid species has different mycorrhizal fungal communities in different habitats [49-52]. A cross-continental scale comparison of the mycorrhizal fungal communities associated with *Gymnadenia conopsea* and *Epipactis helleborine* revealed significant shifts in the fungal community composition of both orchids between China and Europe, and the similarity of their mycorrhizal fungal communities decreased significantly with increasing geographic distance [53].

On the other hand, the germination rates of *Mycena* isolated from the PH, YL, and LF sites showed significant differences, which reflect the unique distribution pattern of certain *Mycena* species with variation in germination. All *Mycena* isolates from the *G. elata* protocorms belong to Clade 1, indicating that *Mycena* species in Clade 1 have a strong ability to germinate *G. elata* seeds, while other clades may lack or have a weaker ability. A study also suggested that seed germination of *G. elata* depends on a narrow group of Mycenaceae fungi [54]. All *Mycena* isolated from PH were OTU4 (*M. polygramma* group), and the LF plot contained 95.83% abundance of OTU2 (unclassified *Mycena* group 1). These *Mycena* strains have not been reported previously to promote the germination of *G. elata* seeds, indicating that the PH and LF sites have high-quality *Mycena* resources for *G. elata* germination. The YL plot may have a lower germination rate due to the presence of OTU1 (*M. citrinomarginata* group) with an abundance of 51.11%. OTU1, OTU3, and OTU4 were assigned to the *M. citrinomarginata* group, *M. abramsii* group, and *M. polygram* group, respectively. The germination rates of OTU2, OTU3, and OTU4 were significantly higher than that of OTU1. This result suggests that there is a correlation between the phylogenetic position of *Mycena* and germination rate. Similarly, a study using in vitro symbiosis between six *Mycena* fungi and *G. elata* seeds showed that the seed germination rates of KFRI1212 and KFRI2121 were 60.1% and 47.0%, respectively, while the germination rates of the other four *Mycena* species were below 3.5% [54]. Molecular identification and phylogenetic analysis indicated that these two fungi belong to the same branch. Five OrM fungi isolated from *Anacamptis papilionacea* were selected to test the efficiency of seed germination. Among them, four belonged to the *Tulasnella calospora* species complex and one belonged to *Ceratobasidium*. The results showed that the *T. calospora* species complex had a higher germination rate [55]. Overall, the *Mycena* resources in the Zhaotong area are rich, and many *Mycena* species have a significantly higher germination rate than the control commercial *Mycena* J3. In this study, two strains (YL10 and YL16) had more effective germination to produce protocorms than the control commercial *Mycena* J3 in a field experiment, suggesting that these two strains have some market application potential.

The symbiotic relationship between orchids and mycorrhizal fungi changes as the plant develops. In *Cephalanthera damasonium* and *Cephalanthera longifolia*, *Piriformospora indica* and *Sebacina vermifera* are the main fungal partners during the seed germination and seedling development stages, whereas *T. calospora* and *Ceratobasidium* sp. dominate in the adult stage [56]. *Cyrtosia septentrionalis* shows a similar pattern, with *Physisporinus* inducing the seed germination stage and *Armillaria* associating with the adult stage [57]. The fungal community associated with *G. elata* also changes with the developmental stages [58]. In this study, most of the *G. elata* tissues from YL were at the protocorm stage of new germination (Fig. S6A), while most of the *G. elata* tissues from LF and PH were at the protocorm stage of elongation (Fig. S6B). The newly germinated protocorms (Fig. S6A) had low fungal diversity and were predominantly colonized by the basidiomycete *Mycena* (90% abundance), supporting the hypothesis that *G. elata* requires *Mycena* symbionts for germination [26]. However, the elongated protocorms (Fig. S6B) exhibited higher fungal diversity and a shift in fungal dominance to the Ascomycetes *Ilyonectria* and *Cadophora*, with *Mycena* abundance decreasing to 11.88% and 10.71%, respectively.

Orchid mycorrhizal fungi are predominantly Basidiomycetes [59, 60], but Shefferson *et al*. [61] also detected Ascomycetes in *Phialophora*. The mycorrhizal fungi that facilitate seed germination and protocorm formation may not sustain the growth of seedlings [61]. Orchids may switch to other fungi that offer greater advantages in resource acquisition. For instance, the fully mycoheterotrophic *G. elata* changed its fungal symbiont from *Mycena* to *Armillaria*, which may allow it to access a larger carbon pool [62]. Instead of switching to the colonization of *Armillaria*, we found that *G. elata* increases the abundance of Ascomycetes during the elongation protocorm stage. One possible reason is to sacrifice functional superiority for survival [63, 64]. Under ecological constraints, plants must associate with any accessible fungal species, regardless of whether they are the optimal partners or not [65]. In the absence of *Armillaria* in the vicinity, we assumed that *G. elata* may replace its fungal symbionts after germination to reduce the risk of death by symbioting with some Ascomycetes (Table 1). Therefore, it is interesting to explore the role of these non-*Mycena* fungi in symbiosis with *G. elata*.

The interaction between orchids and mycorrhizal fungi is also influenced by the genetic background of the orchid host. Different orchid genera or species may symbiose with different fungal groups. For example, the dominant symbiotic fungal group of plants in the genus *Goodyera* is *Ceratobasidium* [66], while the dominant symbiotic fungal group of plants in the genus *Arundina* is *Tulasnella* [67]. The host orchid’s genetic background, which includes its phylogeny, ploidy level and genome composition, may influence the composition of its mycorrhizal fungal community [68-72]. Strains isolated from *G. elata* f. *glauca* and *G. elata* f. *viridis* were different in abundance (Table 1) and *Mycena* composition (Fig. 3B, Table S3). Moreover, the average germination rate for *G. elata* f. *viridis* was higher than that for *G. elata* f. *glauca* (Fig. 4D). These facts reflect that fungi may have different affinities for the genetic characteristics of the *G. elata* variety.

## Conclusion

We used an *in situ* baiting method to characterize the symbiotic fungal community associated with different varieties of *G. elata* in natural habitats. Moreover, we also explored the correlation between the phylogenetic status of the symbiotic fungus *Mycena* and the *G. elata* germination rate. We found that (1) there are diverse *Mycena* in natural habitats in Zhaotong, China. Multigene analysis revealed that they phylogenetically belong to the Basidiospore amyloid group (Clade 1). (2) There is a phylogenetic signal of *Mycena* for germination of *G. elata*. Those strains phylogenetically close to *M. abramsii* (OTU3), *M. polygramma* (OTU4), and an unclassified *Mycena* (OTU2) had significantly higher germination rates than those to *M. citrinomarginata* (OTU1). (3) The *Mycena* distribution depends on geographic site and *G. elata* variety. The YL site harbors OTU1 (*M. citrinomarginata* group) and OTU3 (*M. abramsii* group); the LF site harbors mainly OTU2 (unclassified *Mycena* group 1); and the PH site harbors all strains of OTU4 (*M. polygramma* group). Regarding the two varieties of *G. elata*, both OTU2 (unclassified *Mycena* group 1) and OTU3 (*M. abramsii* group) were dominant; in contrast, OTU1 (*M. citrinomarginata* group) occurred dominantly in *G. elata* f. *glauca* but was absent in *G. elata* f. *viridis*. Our results indicate that the community composition of numerous *Mycena* resources in the Zhaotong area varies by geographical location and *G. elata* variety. Importantly, our results also indicate that *Mycena*’s phylogenetic status is correlated with its germination rate, which is important for understanding the geographic distribution and coevolution of *Mycena*-*G. elata* symbiosis in nature. Moreover, it is also valuable for practical guidance for isolating and obtaining novel germination-promoting *Mycena* resources.

## Supplemental Materials

Supplementary data for this paper are available on-line only at http://jmb.or.kr.



## Figures and Tables

**Table 1:** Geographic distribution and *G. elata* source isolation information for all strains.

	Taxonomic group	Phylum	YL	LF	PH	G	V	Total	Relative abundance
1	*Acremonium*	Ascomycota	0	0	1	0	1	1	0.36%
2	*Cadophora*	Ascomycota	0	71	8	61	18	79	28.21%
3	*Exophiala*	Ascomycota	0	0	1	0	1	1	0.36%
4	*Ilyonectria*	Ascomycota	0	104	12	97	19	116	41.43%
5	*Lachnum*	Ascomycota	0	1	0	1	0	1	0.36%
6	*Mollisia*	Ascomycota	1	0	0	0	1	1	0.36%
7	*Mycena*	Basidiomycota	45	23	0	61	7	68	24.29%
8	*Ophiosphaerella*	Ascomycota	0	1	0	0	1	1	0.36%
9	*Paraphaeosphaeria*	Ascomycota	0	1	0	0	1	1	0.36%
10	Unclassified Mycenaceae	Basidiomycota	0	1	3	4	0	4	1.43%
11	Unclassified Sordariales	Ascomycota	0	0	2	0	2	2	0.71%
12	*Xenopolyscytalum*	Ascomycota	4	0	0	0	4	4	1.43%
13	Unclassified Xylariales	Ascomycota	0	0	1	1	0	1	0.36%
Total			50	202	28	225	55	280	100%

YL, LF, and PH are strains isolated from Yiliang, Lianfeng and Panhe, respectively. G and V are strains isolated from *G. elata* f. *glauca* and *G. elata* f. *viridis*, respectively. The leftmost column exhibits the relative abundance of each taxonomic group.
