# Supplementary material for: Diverse Mycena Fungi and Their Potential for Gastrodia elata Germination
Source: J Microbiol Biotechnol. 2024 Apr 29;34(6):1249–59. doi: 10.4014/jmb.2401.01009 (PMC11239410; doi:10.4014/jmb.2401.01009)
Supplement: Supplementary file 1 [file jmb-34-6-1249-supple.pdf]

J. Microbiol. Biotechnol. 2024. 34(6): 1249–1259

https://doi.org/10.4014/jmb.2401.01009

Supplementary Figures and Tables

Diverse *Mycena* fungi and their potential for *Gastrodia elata* germination

Supplementary figures and tables

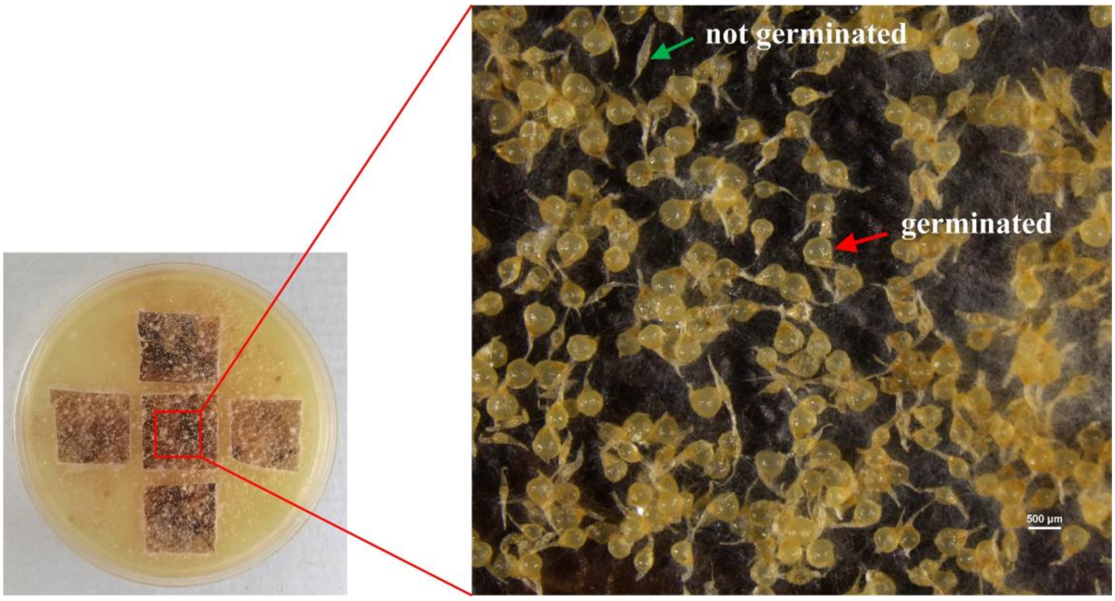

Fig. S1. Photos for a germination experiment. It demonstrated seeds without germination **(green arrow)** and the protocorms that have sprouted from the seeds **(red arrow)**

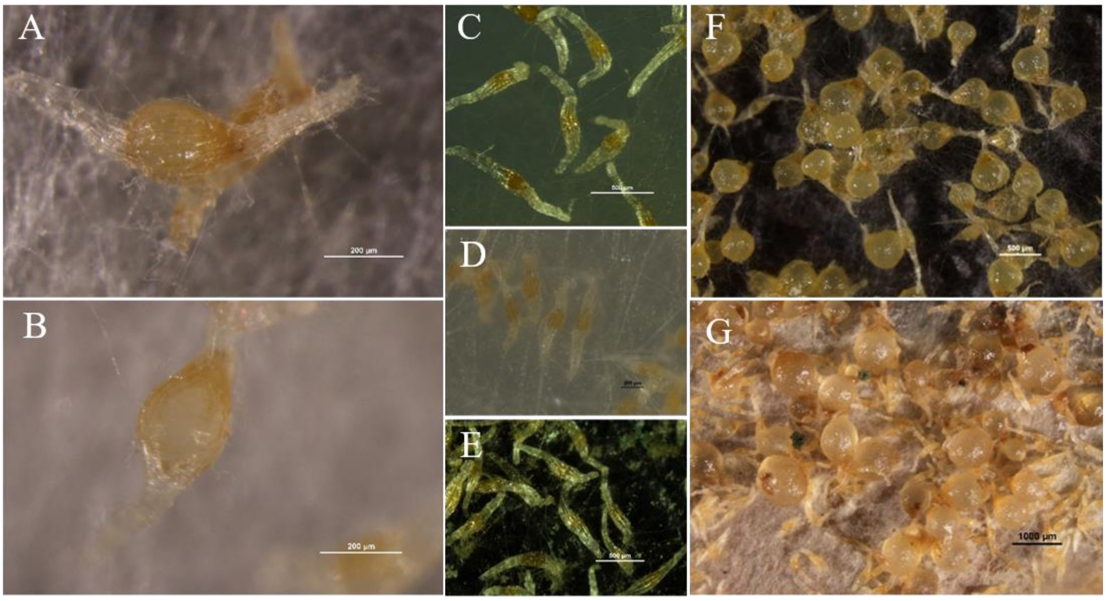

Fig. S2. The effect of *Mycena* and the control on the germination of *G. elata* seeds. Panels A and B show seed coat expansion without rupture under a 100x microscope and seed coat expansion and rupture, respectively; panels C, D and E demonstrate that three negative controls (seeds were sprinkled directly on water agar plates, PDA plates and sterile Fagaceae leaves and placed on water agar plates) did not promote germination; panels F and G illustrate the germination of strain PH30 (*M. polygramma* group) and the control commercial *Mycena* J3 (*M. citrinomarginata* group) under 20x magnification, respectively.

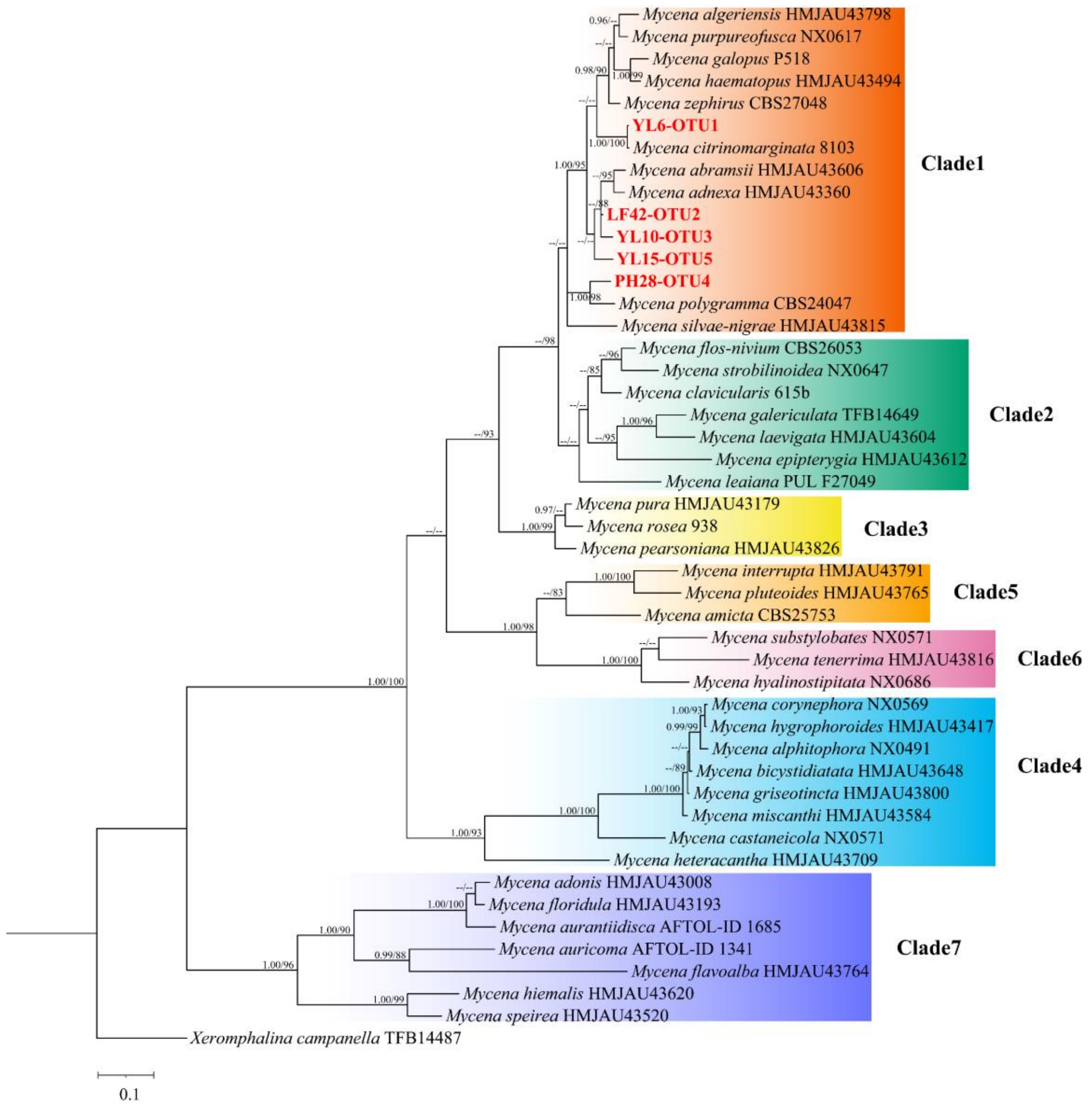

Fig. S3. ***Mycena*** **phylogeny from ITS gene analysis** (BPP ≥ 0.95, Bootstrap ≥ 75% on nodes, – otherwise)**.** Sequences in this study are shown in bold red font, with the symbol "-" followed by the OTU name identified at the 99% similarity level. The tree is rooted with *Xerphalina campanella*.

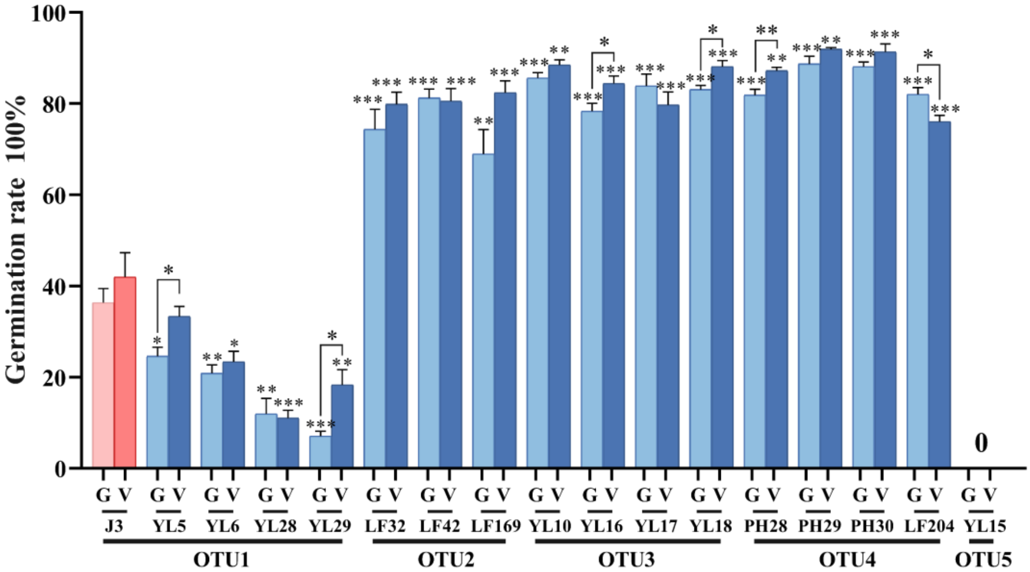

Fig. S4. Statistical analysis of the germination rate of *G. elata* seeds by different *Mycena*. G is the germination rate of *G. elata* f. *glauca*; V is the germination rate of *G. elata* f. *viridis*; J3 is the control commercial *Mycena*. The uppercase letters and numbers on the line where J3 is located represent the *Mycena* strain number. An independent sample T test was used to compare the germination rate between *G. elata* f. *glauca* and *G. elata* f. *viridis* for each strain, as well as the germination rate between each tested strain and the control commercial *Mycena* J3. Asterisks denote statistically significant differences $*P < 0.05$, $**P < 0.01$, $***P < 0.001$. Data are mean ± SEM, $n \geq 3$.

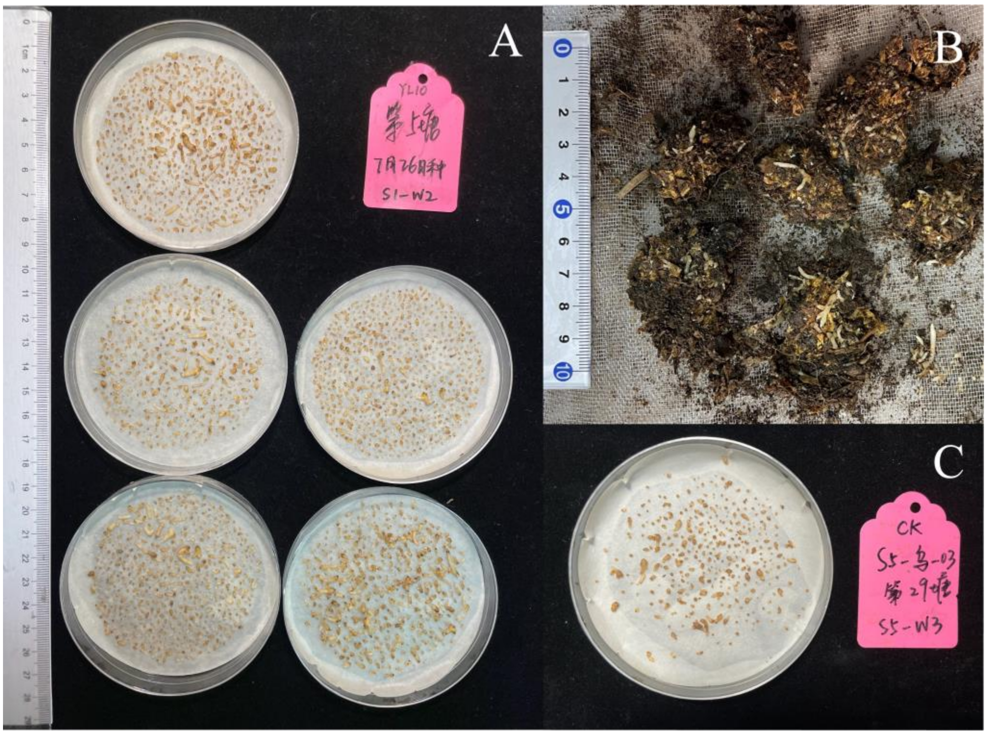

Fig. S5. Germination rate in the field experiment. Fig. A displays the germination of strain YL10 in one treatment; Fig. B demonstrates the actual germination of strain YL10 in a nylon bag; Fig. C exhibits the germination status of the control commercial *Mycena* J3 in one treatment.

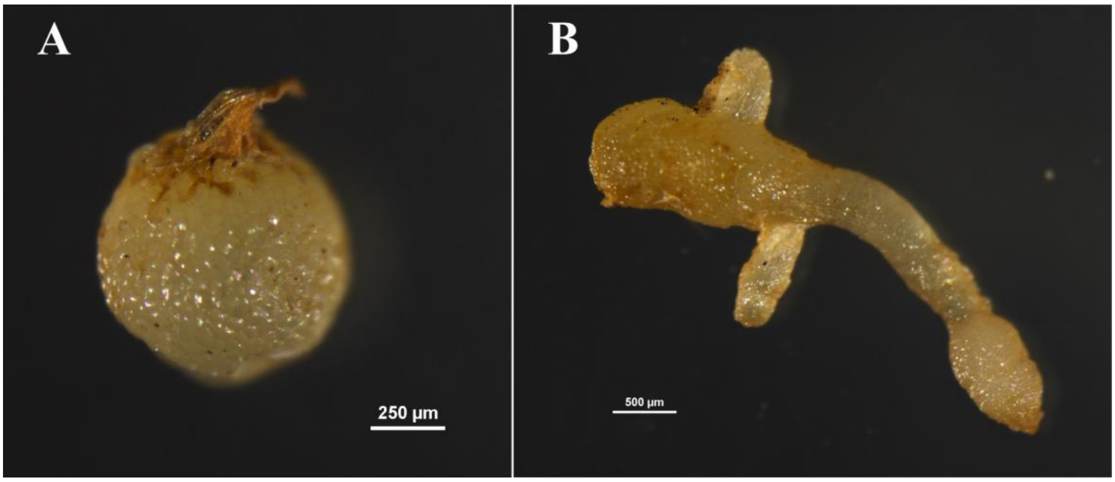

Fig. S6. The newly germinated protocorm of *G. elata* from the YL plot (A) and the elongated protocorms of *G. elata* from the LF and PH plots (B).

Table S1. Taxonomy information of 5 representative *Mycena* strains isolated from *Gastrodia elata* protocorms tissues. The numbers in brackets represent the similarity between our sequences and the sequences in the UNITE database.

Representative strain code	Number of isolate	Relative abundance	Taxonomy	ITS accession numbers	nLSU accession numbers	SSU accession numbers
LF42	23	0.3194	k__Fungi(100);p__Basidiomycota(98);c__Agaricomycetes(98);o__Agaricales(98);f__Tricholomataceae(98);g__Mycena(98);unclassified; unclassified;	OR759509	OR754290	OR763347
PH28	4	0.0556	k__Fungi(100);p__Basidiomycota(97);c__Agaricomycetes(97);o__Agaricales(97);f__Tricholomataceae(97);g__Mycena(97);unclassified; unclassified;	OR759510	OR754291	OR763348
YL10	21	0.2917	k__Fungi(100);p__Basidiomycota(91);c__Agaricomycetes(91);o__Agaricales(91);f__Tricholomataceae(91);g__Mycena(91);unclassified; unclassified;	OR759511	OR754292	OR763349
YL15	1	0.0139	k__Fungi(100);p__Basidiomycota(99);c__Agaricomycetes(99);o__Agaricales(99);f__Tricholomataceae(99);g__Mycena(99);s__Mycena_robusta(92);M	OR759512	OR754293	OR763350

			ycena_robusta(92); Mycena_robusta(92);			
YL6	23	0.3194	k__Fungi(100);p__Basidiomycota(100);c__Agaricomycetes(100);o__Agaricales(100);f__Tricholomataceae(100);g__Mycena(100);unclassified;unclassified;	OR759513	OR754294	OR763351

Table S2. Specimens used in phylogenetic analysis and GenBank accession numbers.

No.	Species	Voucher	GenBank accession no.			Locality	Reference
			ITS	nLSU	SSU		
1	*Mycena abramsii*	HMJAU43606	MH396629	MK629355	MK629336	China	[1]
2	*M. adnexa*	HMAJU43360	MK733290	MK722345	MK722315	China	[2]
3	*M. adonis*	HMJAU43008	MK309769	-	-	China	[2]
4	*M. algeriensis*	HMJAU43798	MK733295	MK722347	-	China	[2]
5	*M. alphitophora*	NX0491	MH136830	-	-	China	[1]
6	*M. amicta*	CBS 257.53	MH857184	MH868722	-	France	[3]
7	*M. aurantiidisca*	AFTOL-ID 1685	DQ490646	DQ470811	DQ457694	America	[4]
8	*M. auricoma*	AFTOL-ID 1341	DQ490647	-	DQ457695	America	[4]
9	*M. bicystidiatata*	HMJAU43648	MK309773	MK629359	MK629341	China	[2]
10	*M. castaneicola*	NX0571	MH136826	-	-	China	[1]
11	*M. citrinomarginata*	8103	OM228752	OM228760	-	China	[5]

12	*M. clavicularis*	615b	JF908467	-	-	Italy	[6]
13	*M. corynephora*	NX0569	MH136833	-	-	China	[1]
14	*M. epipterygia*	HMJAU4 3612	MH396632	-	MK629337	China	[1]
15	*M. flavoalba*	HMJAU4 3764	MT497547	-	-	China	[2]
16	*M. floridula*	HMJAU4 3193	MK309770	-	-	China	[2]
17	*M. flos-nivium*	CBS 260.53	MH857186	MH868724	-	France	[3]
18	*M. galericulata*	TFB1464 9	MN088382	-	-	America	[7]
19	*M. galopus*	P518	MZ078482	-	-	Poland	[8]
20	*M. griseotincta*	HMJAU4 3800	MK309783	MK629363	MK629346	China	[2]
21	*M. haematopus*	HMJAU4 3494	MK733296	MK722351	MK722319	China	[2]
22	*M. heteracantha*	HMJAU4 3709	MK309785	MK629362	MK629345	China	[2]
23	*M. hiemalis*	HMJAU4 3620	MK309790	-	-	China	[2]
24	*M. hyalinostipitata*	NX0686	MH136828	-	-	China	[1]

25	*M. hygrophoroides*	HMJAU43417	MK309780	-	MK629327	China	[9]
26	*M. interrupta*	HMJAU43791	MK733300	-	-	China	[2]
27	*M. laevigata*	HMJAU43604	MK733303	MK722354	MK722322	China	[5]
28	*M. leaiana*	PUL F27049	MW448623	-	-	Poland	Unpublished
29	*M. miscanthi*	HMJAU43584	MK309779	-	MK629335	China	[9]
30	*M. pearsoniana*	HMJAU43826	MK733305	MK722356	-	China	[2]
31	*M. pluteoides*	HMJAU43765	MK733306	-	-	China	[2]
32	*M. polygramma*	CBS 240.47	MH856235	MH867764	-	France	[3]
33	*M. pura*	HMJAU43179	MK309794	-	-	China	[9]
34	*M. purpureofusca*	NX0617	MG654740	-	-	China	[3, 10]
35	*M. rosea*	938	JF908487	-	-	Italy	[6]
36	*M. sanguinolenta*	GLM 45982	-	AY207257	-	Europe	[11]

37	*M. silvae-nigrae*	HMJAU4 3815	MK733310	-	-	China	[2]
38	*M. speirea*	HMJAU4 3520	MT497548	-	-	China	[2]
39	*M. strobilinoidea*	NX0647	MG654743	-	-	China	[12]
40	*M. substylobates*	NX0571	MH216189	-	-	China	[1]
41	*M. tenerrima*	HMJAU4 3816	MK309796	MK629364	-	China	[9]
43	*M. zephirus*	CBS 270.48	MH856339	MH867892		France	[3]
42	*Xeromphalina campanella*	TFB1448 7	KP835678	KM011910	-	America	[10]

Table S3 Statistical analysis of the OTU relative abundance of the *Mycena* sequences at a 99% similarity level in ITS+nLSU+SSU genes.

	OTU	Group	YL	LF	PH	G	V	Total	Relative abundance
YL6	OTU1	*M. citrinomarginata*	23	0	0	23	0	23	31.94%
LF42	OTU2	unclassified_*Mycena*	0	23	0	21	2	23	31.94%
YL10	OTU3	*M. abramsii*	21	0	0	17	4	21	29.17%
PH28	OTU4	*M. polygramma*	0	1	3	4	0	4	5.56%
YL15	OTU5	unclassified_*Mycena*	1	0	0	0	1	1	1.39%
Total			45	24	3	65	7	72	100%

Note: YL, LF, and PH are strains isolated from Yiliang, Liangfeng and Panhe, respectively. G and V are strains isolated from *G. elata* f. *glauca* and *G. elata* f. *viridis*, respectively. The leftmost column exhibits the relative abundance of each genus.

References

1. Na Q, Bau T. 2019. *Mycena* section *Sacchariferae*: three new species with basal discs from China. *Mycological Progress.* **18:** 483-493.
2. Na Q. 2019. Taxonomy and Phylogeny of *Mycena* in China. PhD thesis. Jilin Agricultural University.
3. Vu D, Groenewald M, Vries M, Gehrmann T, Stielow B, Eberhardt U*, et al.* 2019. Large-scale generation and analysis of filamentous fungal DNA barcodes boosts coverage for kingdom Fungi and reveals thresholds for fungal species and higher taxon delimitation. *Studies in Mycology.* **92:** 135-154.
4. Matheny P, Curtis J, Valerie H, Aime M, Moncalvo J-M, Ge Z-W*, et al.* 2006. Major clades of Agaricales: A multilocus phylogenetic overview. *Mycologia.* **98:** 982-995.
5. Liu L-n, Zhou G-y, Shen A-r, Shen B-m, Tan Y, Tan Z-m. 2022. *Mycena subpiligera* sp. nov., a Symbiotic Species from China Associated with the Seed Germination of *Gastrodia elata*. *Mycobiology.* **50:** 294-301.
6. Osmundson T, Robert V, Schoch C, Baker L, Smith A, Robich G*, et al.* 2013. Filling Gaps in Biodiversity Knowledge for Macrofungi: Contributions and Assessment of an Herbarium Collection DNA Barcode Sequencing Project. *PloS one.* **8:** 62419.
7. Hughes K, Matheny P, Miller A, Petersen R, Iturriaga T, Johnson K*, et al.* 2020. Pyrophilous fungi detected after wildfires in the Great Smoky Mountains National Park expand known species ranges and biodiversity estimates. *Mycologia.* **112:** 677-698.
8. Jankowiak R, Stępniewska H, Bilański P, Taerum S. 2022. Fungi as potential factors limiting natural regeneration of pedunculate oak (*Quercus robur*) in mixed-species forest stands in Poland. *Plant Pathology.* **71:** 805-817.
9. Na Q, Bau T. 2019. Recognition of *Mycena* sect. *Amparoina* sect. nov. (Mycenaceae, Agaricales), including four new species and revision of the limits of sect. *Sacchariferae*. *MycoKeys.* **52:** 103-124.
10. Aravindakshan D, Manimohan P. 2015. *Mycenas of Kerala*, pp. 1-223. Ed. SporePrint Books, Calicut, Kerala, India.
11. Walther G, Garnica S, Weiss M. 2005. The systematic relevance of conidiogenesis modes in the gilled *Agaricales*. *Mycological research.* **109:** 525-544.
12. Na QIN, Bau T. 2018. New species of *Mycena* (Mycenaceae, Agaricales) with colored lamellae and three new species records from China. *Phytotaxa.* **361:** 266-278.
